# The Impact of Vitamin D Receptor Gene Polymorphisms (*FokI, ApaI, TaqI*) in Correlation with Oxidative Stress and Hormonal and Dermatologic Manifestations in Polycystic Ovary Syndrome

**DOI:** 10.3390/medicina60091501

**Published:** 2024-09-14

**Authors:** Vulcan Talida, Suciu Sergiu Tudor, Iancu Mihaela, Mitrea Daniela-Rodica, Filip Gabriela A., Procopciuc Lucia Maria

**Affiliations:** 1 Department of Dermatology, Iuliu Hațieganu University of Medicine and Pharmacy, 400006 Cluj-Napoca, Romania; talida.vulcan@umfcluj.ro; 2 Department of Maxillofacial Surgery and Radiology, Iuliu Hațieganu University of Medicine and Pharmacy, 400326 Cluj-Napoca, Romania; suciu.tudor.sergiu@elearn.umfcluj.ro; 3 Medical Informatics and Biostatistics, Department 11—Medical Education, Faculty of Medicine, “Iuliu Hatieganu” University of Medicine and Pharmacy, 400349 Cluj-Napoca, Romania; miancu@umfcluj.ro; 4 Department of Physiology, Iuliu Hațieganu University of Medicine and Pharmacy, 400006 Cluj-Napoca, Romania; rdmitrea@gmail.com; 5 Department of Anatomy and Embriology, Iuliu Hațieganu University of Medicine and Pharmacy, 400006 Cluj-Napoca, Romania; 6 Department of Biochemistry, Iuliu Hațieganu University of Medicine and Pharmacy, 400006 Cluj-Napoca, Romania; lprocopciuc@umfcluj.ro

**Keywords:** acne, hirsutism, polymorphism, VDR, polycystic ovary syndrome

## Abstract

*Background and Objectives*: Polycystic ovary syndrome (PCOS) is a frequent and complex multidisciplinary disorder. Data regarding the role of genes involved in vitamin D metabolism in PCOS are as-yet elusive but suggest an association of VDR (vitamin D receptor) and vitamin D levels with metabolic, endocrine and cutaneous manifestations. The aim of this study was to evaluate the association between VDR gene polymorphisms and cutaneous manifestations, to find a correlation between hormonal parameters, oxidative stress and skin manifestations in women with PCOS, and to determine the impact of VDR gene polymorphisms on these parameters. *Materials and Methods*: This case–control study included 39 controls and 46 women with PCOS, matched by age and BMI distribution. Acne, hirsutism, seborrhea, androgenetic alopecia, oxidative stress and androgen hormones were recorded. VDR gene polymorphisms ApaI, FokI and TaqI were examined by polymerase chain reaction restriction fragment length polymorphism, and the androgen hormone (total testosterone, DHEAS), SHBG and malondialdehyde levels were assessed. *Results*: The most frequent skin manifestations in PCOS cases were acne followed by seborrhea, hirsutism and androgenic alopecia. The VDR-FokI polymorphism CC genotype had a significant protective role in the odds of acne (OR = 0.11, 95% CI: [0.02, 0.70], *p* = 0.015, p-corrected = 0.040) and seborrhea (OR = 0.15, 95% CI: [0.03, 0.75], *p* = 0.019, p-corrected = 0.039). The results demonstrated a significant protective effect of the C allele on the odds of acne and seborrhea in PCOS cases. Moreover, the dominant genotype of VDR-TaqI could have a protective role against oxidative stress (lower MDA levels) compared to patients carrying the TT genotype. *Conclusions*: In summary, this is the first study to demonstrate that the FokI CC genotype may have a protective role against both acne and seborrhea in women with PCOS, while the VDR-TaqI dominant genotype is associated with diminished oxidative stress in PCOS patients.

## 1. Introduction

Polycystic ovary syndrome (PCOS) is a frequent and complex multidisciplinary disorder that commonly occurs in reproductive-age females. PCOS affects 10–13% of women of reproductive age and has a wide variety of clinical manifestations including skin signs of hyperandrogenism (acne, seborrhea, hirsutism, androgenetic alopecia) associated with oligo/anovulation and polycystic ovary morphology [1]. Positioned at the interface between the skin, endocrine and reproduction system, dermatologists play a central role in the identification and diagnosis of this disease, frequently being the first ones that patients seek the services of, due to skin changes and hirsutism that cause a decrease in the quality of life.

It is known that PCOS is associated with an increased risk of type 2 diabetes mellitus, cardiovascular disease and metabolic syndrome [2] due to hyperinsulinemia and insulin resistance, obesity and hormonal metabolism abnormalities, as the Rotterdam ESHRE/ASRM-Sponsored PCOS Consensus Workshop Group stated in 2003 and revised last time in 2023 [3]. Thus, in PCOS, there are disturbances in the metabolism of estrogens and androgens [4], the hypothalamic–pituitary–ovarian axis works abnormally and the synthesis of androgen is increased in the cells of internal theca of the ovaries [5]. This process is stimulated by LH secreted in excess under GnRH, while FSH increases the activity of aromatase in granulosa cells and both hormones lead to high levels of androgens. Hyperandrogenemia triggers follicular maturation and leads to an altered response of gonadotrophins [4].

Besides hyperandrogenism, dyslipidemia and obesity represent an important source of free radicals in PCOS, and their excessive generation leads to redox imbalance, lipid peroxidation and insulin resistance [6]. The uptake of glucose in muscular and adipose tissues is altered, and the secretion of insulin in pancreatic β cells is affected. Additionally, endothelial dysfunction occurs due to cell membrane damage and the impairment of nitric oxide secretion [7], and atherosclerosis and cardiovascular diseases will develop [8].

Several studies have demonstrated that vitamin D controls different endocrine processes including calcium–phosphate homeostasis [9] and bone metabolism and influences the regulatory mechanisms of female reproductive cycles [10]. Vitamin D modulates the expression of human chorionic gonadotropin hormone, increases the production of female hormones [11] and, in low concentrations, is associated with follicular arrest and infertility in PCOS [12]. In turn, insulin resistance and obesity influence the vitamin D levels in blood in PCOS [13], and its administration improves insulin sensitivity, glucose homeostasis and metabolic syndrome [14] in PCOS.

Vitamin D binds to vitamin D receptor (VDR), leading to the transcription of vitamin D-related genes [15]. Data regarding the role of gene variants involved in vitamin D metabolism in PCOS are as-yet elusive but suggest an association of VDR and vitamin D levels with metabolic, endocrine and cutaneous manifestations in women with PCOS. Previous studies have suggested that VDR gene variants are also associated with susceptibility to PCOS and the severity of the phenotype. However, the role of the VDR gene is still inconsistent, and the results vary among different populations due to the polymorphic trait of the VDR gene and the variability among different races of genotype/allele frequency. The clinical manifestations are thought to be consecutive to androgen excess, which leads to dermatological and gynecological disturbances. Specific cutaneous features include hirsutism, acne, seborrhea, androgenic alopecia and acanthosis nigricans. Hirsutism is frequently used as an indicator of hyperandrogenism, being, in some studies, reported as the most common skin manifestation in PCOS and closely related to metabolic abnormalities [16].

Based on these data, the objective of this work is to provide new insights regarding the associations of VDR polymorphisms *FokI*, *TaqI* and *ApaI* with PCOS and to demonstrate a significant correlation between skin and hormonal changes in PCOS and VDR polymorphisms.

## 2. Materials and Methods

In this prospective study, 46 women with PCOS and 39 control women within the same age and BMI range were enrolled. Women with PCOS were recruited from the outpatient section of the Department of Dermatovenerology, County Hospital, Cluj-Napoca, Romania, or were referred to our clinic by gynecologists for adequate treatment of cutaneous lesions. The ultrasound examinations of ovaries were performed using a Siemens G60 Ultrasound machine with an appropriate transducer (3.5 MHz), in the Gynecology Department of County Hospital, Cluj Napoca, Romania. Informed consent was obtained from all participants (Hospital Agreement 38.830/20 November 2020).

PCOS was diagnosed according to the Rotterdam consensus of two of the following three findings: clinical/biochemical hyperandrogenism, chronic ovulatory dysfunction and polycystic ovarian morphology [2,3]. Patients with underlying conditions that cause hyperandrogenemia or ovarian dysfunction (pregnancy, ovarian or androgen-secreting tumor, thyroid dysfunction, hyperprolactinemia, Cushing syndrome, congenital adrenal hyperplasia or hormonal medication) were excluded. Patients with PCOS or controls with other causes of oligomenorrhea or impaired renal or hepatic function were also excluded from this study. The patients did not take any medication known to affect endocrine, metabolic or hormonal parameters for at least 3 months before entering the study. All the PCOS participants were eligible for inclusion regardless of their phenotype, because the sub-grouping of patients would have resulted in a very small sample size per group, limiting the clinical significance. In order to determine the size of samples, a formula related to case–control studies for genetic association studies using the “genpwr” R package was applied. Sample size estimation for a case–control study was based on the desired power (0.80), proportion of cases in the sample (0.5), minor allele frequencies (0.20) and odds ratios to detect (4), and the genetic model used in testing was a dominant model, as previously was published. The total number of subjects required was equal to 76. Cases and controls were selected using convenience sampling (as a non-probability sampling method).

Body mass index (BMI = kg/m^2^) was calculated for every participant [7].Obesity was defined as BMI ≥ 25 kg/m^2^ [17]. Blood samples were obtained on the second–third day of spontaneous menstruation or during the period of amenorrhea, between 08:00 and 09:00 a.m., after overnight fasting, for the assessment of baseline levels of total testosterone (>0.68 ng/mL), sex hormone-binding globulin (SHBG >128 nmol/L) and dehydroepiandrosterone sulfate (DHEA-S > 5.39 µg/mL) by ELISA and malondialdehyde (MDA) by fluorimetry [18].

The subjects were examined regarding the presence of acne, hirsutism, seborrhea and hair loss due to androgenetic alopecia by the same dermatologist. Acne severity was assessed using the Global Acne Grading System (GAGS), investigating six areas of the body (forehead, left and right cheek, nose, chin, chest and upper back) and five types of lesions (no lesion, comedones, papules, pustules, nodules). The severity of acne was graded as mild (score = 1–18), moderate (score = 19–30), severe (score = 31–38) and very severe (score ≥ 39) [19]. Hirsutism, meaning excessive growth of terminal hair, was defined by the modified Ferriman and Gallwey score. The scoring system involves the visual grading of hair growth over nine androgen-sensitive areas, with a score of 8 or more being considered abnormal [20]. Androgenetic alopecia, a diffuse nonscarring type of hair loss in the frontal, central and parietal areas of the scalp, was evaluated according to Ludwig’s classification as grade I or mild, grade II or moderate and grade III or severe [21]. The presence of greasy or oily skin on the face and scalp were defined as seborrhea and it was assessed by analyzing the patient’s answers to several questions about high sebum secretion.

In addition, 2 mL of venous blood was drawn in anticoagulant tubes with EDTA to be used for the detection of VDR-ApaI, VDR-FokI and VDR-TaqI gene polymorphisms by Restriction Fragment Length Polymorphism (PCR RFLP). High-molecular-weight DNA was isolated from leukocytes using a Zymoresearch kit (Quick-DNA Miniprep, Kit-Zymo Research Corporation, Freiburg, Germany) and stored at −20 °C until PCR analysis.

## 3. Statistical Analysis

Quantitative continuous variables were summarized as the arithmetic mean and standard deviation for data with normal distribution or the geometric mean for Log-normal distribution of data. A comparison of hormonal parameters between PCOS and non-PCOS groups was made using Student’s *t*-test or Welch’s *t*-test for independent samples. Differences in frequencies of qualitative clinical characteristics between PCOS and non-PCOS groups were compared using the Chi-square test or Fisher’s exact test. 

The departure from the Hardy–Weinberg equilibrium (HWE) for studied SNPs was tested using the exact Chi-square test from the “genetics” R package. 

The associations between vitamin D receptor gene polymorphisms, hormonal characteristics and oxidative stress and odds of dermatological manifestations (acne, seborrhea, hirsutism) stratified by PCOS and non-PCOS groups were tested by unconditional binomial logistic regression models. The effect size of association was described using the unadjusted odds ratio (OR) with a 95% confidence interval (95% CI). Logistic regression models were applied under four different inheritance genetic models (codominant, dominant, recessive and log-additive) using the R-project package “SNPassoc” version 2.1.0. In order to control the false discovery rate (FDR) when performing multiple genetic models tested, we reported the significance estimated level (p) corrected for multiple comparisons using Benjamini-Hochberg (BH) method.

Associations of vitamin D receptor gene polymorphisms with hormonal characteristics and oxidative stress characteristics in women with PCOS and women without PCOS were tested using parametric tests (Student-t test with equal variances) or nonparametric test (Mann-Whitney).

All statistical analysis was performed in R software, version 4.2.3, R Foundation for Statistical Computing, Vienna, Austria and the results were considered as statistically significant at *p* < 0.05.

## 4. Results

The prevalence of cutaneous features, hormonal status and oxidative stress of women with PCOS and non-PCOS are presented in Table 1. The studied groups were similar in terms of age distribution (*p* = 0.611, range values: 23–30 years in women without PCOS and 20–32 years in women with PCOS) and BMI (geometric mean (GSD): 20.99 (1.15) in women without PCOS; 22.20 (1.16) in women with PCOS, *p* = 0.0838).

The frequencies of cutaneous characteristics were significantly different in patients with PCOS and those without PCOS (Table 1), with higher values in women with PCOS. The most frequent cutaneous manifestations in PCOS cases were acne followed by seborrhea, hirsutism, and androgenic alopecia (40 cases (87%) vs. 37 cases (80.4%) vs. 27 (58.7%) vs. 10 (21.7%)). Regarding hormonal characteristics, we found a significant difference in terms of SHBG (*p* = 0.0128) and DHEA-S (*p* = 0.0121). All women with PCOS were found to have significantly different MDA levels compared with women without PCOS (*p* < 0.0001).

### 4.1. Distribution of Genotypes of VDR-ApaI, VDR-Fokl and VDR-Taql Gene Polymorphisms

The genotype frequencies of VDR-ApaI, VDR-Fokl and VDR-Taql gene polymorphisms were in agreement with the Hardy–Weinberg equilibrium in the women-without-PCOS group (VDR-ApaI: p_HWE_ = 1.000, VDR-Fokl: p_HWE_ = 1.000, VDR-Taql: p_HWE_ = 0.719).

### 4.2. Association of Vitamin D Receptor Gene Polymorphisms, Hormonal Characteristics and Oxidative Stress with Dermatological Manifestations

The effects of VDR-ApaI, VDR-FokI and VDR-TaqI gene polymorphisms on hormonal, oxidative stress and clinical cutaneous manifestations parameters in cases of women with PCOS and control women were analyzed by codominant, dominant (AC + AA vs. CC for VDR-ApaI, CT+CC vs. TT for VDR-FokI, CT+TT vs. TT for VDR-TaqI), recessive (AA vs. CC+AC for VDR-ApaI, CC vs. CT+TT for VDR-FokI, TT vs. CT+CC for VDR-TaqI) and allelic genetic models. The results of logistic regression applied to the codominant genotype model showed a significant difference in the genotypic distribution (TT, CT and CC) of the VDR-Fokl gene polymorphism between PCOS cases with acne and without acne (*p* = 0.034, p-corrected = 0.044) but no significant association between genotypes and acne in women without PCOS (*p* = 0.215). Also, in the recessive genotype model (CC vs. CT+TT), we found that the CC genotype had a significant protective role in the odds of acne in the PCOS group (OR = 0.11, 95% CI: [0.02, 0.70], *p* = 0.015, p-corrected = 0.040), while in the non-PCOS group, we found no significant association (*p* = 0.092). We found the T allele of the VDR-Fokl gene polymorphism in 52.5% and 16.7% and the C allele in 47.5% and 8.3% of PCOS cases with acne and PCOS cases without acne, respectively. The results demonstrated a significant protective effect of the C allele on the odds of acne in PCOS cases (OR = 0.18, 95% CI: [0.04, 0.88]). We found no significant difference in allele frequencies in the non-PCOS group (*p* = 0.126). These results showed that there was an interaction between polycystic ovary syndrome and the VDR-FokI gene polymorphism regarding the odds of acne.

There was no significant difference in terms of clinical hormonal parameters (testosterone, SHBG, DHEAS) between the PCOS group with and the PCOS group without acne (Table 2).

The hormonal and oxidative stress characteristics of PCOS cases with and without hirsutism are presented in Supplementary Table S1.

Although we observed slightly higher values of testosterone, SHBG, DHEAS and MDA levels in PCOS patients with hirsutism compared with PCOS cases without hirsutism, the differences were not statistically significant (*p* > 0.05).

The results of logistic regression applied to the codominant genotype model showed a significant difference in the genotypic distribution (TT, CT and CC) of the VDR-Fokl gene polymorphism between PCOS cases with seborrhea and without seborrhea (*p* = 0.033, p-corrected = 0.044) but no significant association between genotypes and seborrhea in women without PCOS (*p* = 0.807) (Table 3). Also, in the recessive genotype model (CC vs. CT+TT), we found that the CC genotype had a significant protective role in the odds of seborrhea in the PCOS group (OR = 0.15, 95% CI: [0.03, 0.75], *p* = 0.019, p-corrected = 0.039), while in the non-PCOS group, we found no significant association (*p* = 0.358). Also, the allelic model demonstrated a significant protective effect of the C allele on the odds of seborrhea in PCOS cases (OR = 0.24, 95% CI: [0.07, 0.81]), while in the non-PCOS group, we found no significant difference in allele frequencies (*p* = 0.454). These results showed that polycystic ovary syndrome has a modifying effect on the association between the VDR-FokI gene polymorphism and seborrhea.

The hormonal and oxidative stress characteristics of PCOS cases with and without androgenic alopecia are presented in Supplementary Table S2. Although we observed slightly higher values of testosterone, DHEAS and MDA levels in PCOS patients with alopecia compared with PCOS cases without, the differences were not statistically significant (*p* > 0.05).

### 4.3. Association of Vitamin D Receptor Gene Polymorphisms with Hormonal Characteristics and Oxidative Stress Characteristics

No significant differences were revealed in the testosterone, SHBG and DHEAS between PCOS patients with the dominant genotype of VDR gene polymorphisms and PCOS patients with the wild genotype (Table 4). However, we noticed that the MDA concentrations were lower in PCOS patients with the dominant genotype of VDR-TaqI than in PCOS patients carrying the TT genotype, but the observed difference had marginal significance (*p* = 0.054). Moreover, the difference in MDA concentrations reached statistical significance in the non-PCOS group (*p* = 0.016); the women without PCOS carrying the dominant genotype (CT+CC) had higher MDA values compared with women with the wild genotype (Table 4).

## 5. Discussion

Cutaneous manifestations of PCOS occur early in the course of the disease and play an important role in initial diagnosis. Also, among others, skin features in particular affect the quality of life and psychological well-being of patients [22]. Cutaneous signs in PCOS include acne, hirsutism, seborrhea, androgenetic alopecia and acanthosis nigricans and are usually considered a sign of hyperandrogenism, but the development of skin features is complex. Indeed, androgens play an important role, but as was showed previously in the literature, the clinical manifestations can be observed even in the absence of biochemical hyperandrogenism [23].

A number of studies have shown that acne is the most common dermatological manifestation [24,25]. Our research was consistent with the findings of these studies, with acne being the most frequent skin manifestation in PCOS cases, followed by seborrhea and hirsutism. Hirsutism is used as an indicator of hyperandrogenism. Although it was previously reported as the most common skin manifestation [2,26], acne followed by seborrhea was more common in our report. Moreover, hirsutism in women with PCOS was not correlated with the level of androgens. This can be explained by the fact that hirsutism results from the interaction between the androgen level and the sensitivity of the hair follicle to androgens; therefore, the severity of hirsutism does not always correlate with the androgen levels, because the response of the androgen-dependent follicle to androgen excess varies among persons [24]. Moreover, it is known that serum testosterone circulates bound to SHBG or albumin, so only free testosterone is metabolically and endocrinologically active, and the hormone is more sensitive for the diagnosis of hyperandrogenic conditions. In our study, the values of total testosterone were similar in the PCOS group and controls but SHBG values decreased in those with PCOS, suggesting that in PCOS, a small proportion of the total testosterone is bound to proteins and a larger proportion is free as active testosterone. Androgenetic alopecia was most frequent in the PCOS group, results consistent with other studies [23,25]. Although we observed higher values of androgens in the PCOS group, the differences were not statistically significant, with androgenetic alopecia, like acne and hirsutism, being a poor indicator of biochemical hyperandrogenism in our study, which are results in accordance with other literature reports [23,24,25]. One explanation is the individual response of the pilosebaceous unit to androgen levels, ethnic variations, skin type or skin area [27]. Besides androgen, insulin and insulin-like growth factor-1 can contribute to hair follicle growth and also hyperinsulinemia, which suppress the sex hormone-binding globulin, a modulator of testosterone bioavailability [28]. Androgens, excessively secreted in PCOS, including testosterone, androstenedione, DHEA and DHEAS, cause the premature development of ovarian follicles, form multiple antral follicles and determine anovulation. Their bioavailability depends on the sex hormone-binding globulin (SHBG) protein level in blood.

This is the reason for the evaluation of the total testosterone, DHEAS and SHBG levels in correlation with VDR polymorphisms and oxidative stress. In our study, no significant differences were found in the total testosterone, SHBG and DHEAS between PCOS patients with the dominant genotype of VDR gene polymorphisms and PCOS patients with the wild genotype.

Regarding the association of VDR polymorphisms and hormonal or dermatological manifestation, to our knowledge, this is the first study to demonstrate the interplay of the VDR-FokI polymorphism and cutaneous manifestations in PCOS. Among PCOS cases, in the recessive genotype model, we found that the CC genotype had a significant protective role in the odds of acne and seborrhea, and the protective role of the C allele was observed in this group. Therefore, the results showed the interaction between PCOS and the VDR-FokI polymorphism regarding the odds of acne and seborrhea.

The results regarding the association of VDR gene polymorphisms and androgen levels are contradictory. In our study, the two parameters are not associated, whereas De Song et al. [15] showed that the FokI AG genotype was significantly associated with increased levels of total testosterone. Additionally, in Austrian women, the VDR-ApaI AA genotype was associated with lower testosterone levels [29]. In contrast to this, another study reported that Indian women with the FokI TT genotype presented increased risks for infertility, acne and alopecia [30].

The TaqI genotype/allele distribution pattern in our study did not yield significant results, similar to previous reports [28] which are in contrast to a study performed on Egyptian women, showing that those with the TaqI CC and TC genotypes had higher testosterone and DHEA-S levels [31].

One recent study was focused on the associations between VDR-ApaI and VDR-TaqI polymorphisms and acne vulgaris. The authors detected a significant decrease in the ApaI A allele and AATT combined genotype in PCOS cases compared to controls and a significant increase in the TaqI tt genotype and t allele compared to controls, considering the role of polymorphisms in the pathogenesis of acne, where the A allele and AATT combined genotype could be protective factors against acne development [32].

Oxidative stress is a complex process, resulting from a disequilibrium between pro-oxidants and antioxidants. Numerous studies have shown that markers of oxidative stress increase in blood in patients with PCOS, with the redox imbalance being an important factor in the pathogenesis of PCOS. MDA is the most known product of lipid peroxidation, and its elevated values are associated with metabolic syndrome, oxidative stress, atherosclerosis and diabetes mellitus [33]. In a recent study, MDA values were significantly increased in PCOS groups, especially in obese groups, while the antioxidant parameters decreased. Oxidative stress and an inefficient antioxidant defense in PCOS were correlated with hyperinsulinemia, hypertension and dyslipidemia findings, supporting the concept that oxidative stress is involved in the pathophysiology of PCOS [34]. Moreover, the free radical production is increased in serum and follicular fluid and leads to poor-quality oocytes and consequently to infertility [35]. Additionally, abdominal adiposity is an important source of androgen hormones, adiponectin, cytokines and free radicals which promote inflammation and oxidative stress [36]. ROS induce protein oxidation, lipid peroxidation and DNA damage; affect endothelial cells; and trigger endothelial dysfunction and cardiovascular diseases [37]. Oxidative stress is related to metabolic syndrome and, by the activation of NF-KB, induces the transcription of TNF-α and creates an inflammatory environment, a triggering factor for insulin resistance and hyperandrogenism [38]. All these factors are intricated and lead to the development of complications in PCOS, metabolic syndrome and infertility. In fact, ROS play an important physiological role in ovarian homeostasis and are essential for normal ovarian function. When they are excessively produced, the mitochondria generate more ROS during the oxidative phosphorylation process and lead to cellular damage and sometimes to allostatic loads. Besides this, hyperglycemia, via NADPH oxidase and due to the stimulative effect on TNF-α realized from monocytes, can contribute to ROS generation [39]. In our study, MDA concentrations were lower in PCOS patients with the dominant genotype of VDR-TaqI compared to the TT genotype, and MDA might be seen as a protective factor against redox imbalance associated with PCOS.

A special relationship exists between cortisol, hypothalamic–pituitary–adrenal (HPA) axis activation and vitamin D levels in PCOS. Pasquali et al. [40] described that the activation of the HPA axis in PCOS is associated with the alteration in cortisol pathways, even in normal cortisolemia. The changes in enzyme activities involved in steroid metabolism can probably increase the production of cortisol and androgens in PCOS. Additionally, the imbalance in cortisol secretion is responsible for depression, stress, anxiety, digestive symptoms, sleep deprivation and weight gain, common clinical manifestations seen in PCOS. Therefore, supplementation with vitamin D may improve the metabolic profile, androgen levels or inflammatory status, but the results are sometimes contradictory and depend on the dose used, pharmaceutical formulations, the duration of treatment or the baseline status of vitamin D [41]. Some limitations in the present study should be considered. A potential limitation is the modest sample size. Another limitation is the lack of information on vitamin D status in all 85 subjects, which could have given clarification as to whether supplements of vitamin D impact acne or seborrhea status. Moreover, the interaction of VDR polymorphisms with some factors including diet, living habits and family history and their influence on hormone levels and PCOS development might complete the pathogenetic framework in PCOS and would allow for a more in-depth understanding of the mechanisms involved.

## 6. Conclusions

In summary, the most frequent skin manifestations in patients with PCOS were acne followed by seborrhea, hirsutism and androgenic alopecia. The study demonstrated that the *FokI* CC genotype may have a protective role against both acne and seborrhea in women with PCOS, and also, the VDR-*TaqI* dominant genotype may have a protective role against oxidative stress in PCOS patients. Moreover, although the studied group was small, the population sample was homogeneous and the results obtained were conclusive and demonstrated the interaction between VDR gene polymorphisms and skin signs in PCOS. However, further studies with larger sample sizes are needed to confirm these findings and to establish the exact role of vitamin D and polymorphisms of VDR in the pathogenesis and treatment of PCOS.

## Figures and Tables

**Table 1:** Cutaneous, hormonal and oxidative stress characteristics of women with and without PCOS.

Characteristics	Women without PCOS (n_1_ = 39)	Women with PCOS (n_2_ = 46)	*p*-Value
GAGS score ^(1)^	3 [0; 3]	10.5 [3, 15]	<0.0001 *
Acne, n (%)	25 (64.1)	40 (87.0)	0.0133 *
Severity of acne, n (%)			0.0094 *
Mild	25 (64.1)	36 (78.3)	
Moderate	0 (0.0)	4 (8.7)	
Hirsutism score ^(1)^	0 [0; 2]	9 [4, 12]	<0.0001 *
Hirsutism, n (%)	0 (0.0)	27 (58.7)	<0.0001 *
Seborrhea, n (%)	18 (46.2)	37 (80.4)	<0.0001 *
Androgenic alopecia, n (%)	0 (0.0)	10 (21.7)	0.0015 *
Testosterone total (nmol/L) ^(2)^	1.47 (0.49)	1.44 (0.42)	0.7073
SHBG (nmol/L) ^(3)^	61.62 (1.52)	47.97 (1.62)	0.0128 *
DHEA-S (lg/dL) ^(1)^	0.98 [0.79, 1.19]	1.17 [0.99, 1.45]	0.0121 *
MDA (nmol/mL) ^(1)^	1.24 [1.15, 1.36]	1.90 [1.63, 2.29]	<0.0001 *

^(1)^ GAGS: Global Acne Grading System; SHBG: hormone-binding globulin; DHEA-S: dehydroepiandrosterone sulfate; MDA: malondialdehyde oxidative stress; data presented as ^(1)^ median [25th percentile, 75th percentile] or ^(2)^ arithmetic mean (sample standard deviation) or ^(3)^ geometric mean (geometric standard deviation) or n = number of subjects; *p*-values obtained from Mann–Whitney test, Chi-squared test, Fisher’s exact test or Student’s *t*-test with equal variances applied on transformed data on logarithmic scale; * significant result: *p*-value < 0.05.

**Table 2:** Demographic, hormonal and oxidative stress characteristics, genotypes and alleles of vitamin D receptor gene polymorphisms (FokI, ApaI, TaqI) in the studied groups stratified by acne.

Characteristics	PCOS without Acne (n_1_ = 6)	PCOS with Acne (n_2_ = 40)	*p*-Value	Non-PCOS without Acne (n_1_ = 14)	Non-PCOS with Acne (n_2_ = 25)	*p*-Value
Age (years) ^(a)^	25.83 (3.06)	25.00 (2.68)	0.489	26.71 (2.33)	24.64 (1.73)	0.003 *
BMI (kg/m^2^) ^(b)^	21.39 (1.08)	22.32 (1.17)	0.525	20.21 (1.12)	21.45 (1.16)	0.167
Testosterone total (nmol/L) ^(a)^	1.26 (0.23)	1.46 (0.44)	0.266	1.28 (0.43)	1.58 (0.49)	0.058
SHBG (nmol/L) ^(b)^	44.85 (1.50)	48.44 (1.64)	0.719	62.79 (1.59)	60.96 (1.49)	0.835
DHEA-S (lg/dL) ^(c)^	1.09 [0.87, 1.58]	1.17 [1.01, 1.40]	0.781	0.82 [0.67, 0.94]	1.06 [0.92, 1.27]	0.463
MDA (nmol/mL) ^(c)^	1.73 [1.59, 1.85]	1.99 [1.63, 2.33]	0.138	1.30 [1.18, 1.48]	1.23 [1.09, 1.30]	0.219
*VDR-ApaI genotypes*			0.655			0.845
CC	0 (0.0)	6 (15.0)		5 (35.7)	7 (28.0)	
AC	4 (66.7)	27 (67.5)		6 (42.9)	13 (52.0)	
AA	2 (33.3)	7 (17.5)		3 (21.4)	5 (20.0)	
AC+AA vs. CC	6 (100.0)	34 (85.0)	0.579	9 (64.3)	18 (72.0)	0.619
Alleles			0.318			0.789
C	4 (33.3)	39 (48.8)		16 (57.1)	27 (54.0)	
A	8 (66.7)	41 (51.2)		12 (42.9)	23 (46.0)	
*VDR-Fokl genotypes*			0.034 *			0.215
TT	0 (0.0)	9 (22.5)		5 (35.7)	13 (52.0)	
CT	2 (33.3)	24 (60.0)		6 (42.9)	11 (44.0)	
CC	4 (66.7)	7 (17.5)		3 (21.4)	1 (4.0)	
CT+CC vs. TT	6 (100.0)	31 (77.5)	0.327	9 (64.3)	12 (48.0)	0.325
Alleles						
T	2 (16.7)	42 (52.5)	0.020 *	16 (57.1)	37 (74.0)	0.126
C	10 (8.3)	38 (47.5)		12 (42.9)	13 (26.0)	
VDR-TaqI *genotypes*			1.000			0.610
CC	0 (0.0)	5 (12.5)		2 (14.3)	3 (12.0)	
CT	3 (50.0)	20 (50.0)		7 (50.0)	9 (36.0)	
TT	3 (50.0)	15 (37.5)		5 (35.7)	13 (52.0)	
CT+TT	6 (100.0)	35 (87.5)	1.000	12 (85.7)	22 (88.0)	0.839
Alleles			0.527			0.404
C	3 (25.0)	30 (37.5)		11 (39.3)	15 (30.0)	
T	9 (75.0)	50 (62.5)		17 (60.7)	35 (70.0)	

SHBG: hormone-binding globulin; DHEA-S: dehydroepiandrosterone sulfate; MDA: malondialdehyde oxidative stress; data presented as ^(a)^ arithmetic mean (sample standard deviation) or ^(b)^ geometric mean (geometric standard deviation) or ^(c)^ median [25th percentile, 75th percentile] or n = number of subjects; *p*-values obtained from Mann–Whitney test, Chi-squared test, Fisher’s exact test, Student’s *t*-test with equal variances applied on transformed data on logarithmic scale or nonparametric ANCOVA; * significant result: *p*-value < 0.05.

**Table 3:** Demographic, hormonal and stress oxidative characteristics, genotypes and alleles of vitamin D receptor gene polymorphisms (FokI, ApaI, TaqI) in PCOS patients with and without seborrhea.

Characteristics	PCOS without Seborrhea (n_1_ = 9)	PCOS with Seborrhea (n_2_ = 37)	*p*-Value	Non-PCOS without Seborrhea (n_1_ = 21)	Non-PCOS with Seborrhea (n_2_ = 18)	*p*-Value
Age (years) ^(a)^	26.00 (2.65)	24.89 (2.72)	0.276	25.38 (2.27)	25.39 (2.15)	0.991
BMI (kg/m^2^) ^(b)^	20.99 (1.09)	22.50 (1.17)	0.218	20.55 (1.13)	21.53 (1.17)	0.308
Testosterone total (nmol/L) ^(a)^	1.32 (0.23)	1.46 (0.46)	0.203	1.47 (0.48)	1.48 (0.51)	0.981
SHBG (nmol/L) ^(b)^	40.00 (1.72)	50.12 (1.59)	0.274	63.84 (1.53)	59.12 (1.51)	0.574
DHEA-S (lg/dL) ^(c)^	0.94 [0.65, 1.24]	1.19 [1.03, 1.47]	0.108	0.92 [0.77, 1.09]	1.04 [0.91, 1.25]	0.190
MDA (nmol/mL) ^(c)^	1.83 [1.62, 1.92]	1.97 [1.63, 2.33]	0.524	1.29 [1.18, 1.42]	1.19 [1.08, 1.28]	0.118
*VDR-ApaI genotypes*			0.637			0.197
CC	0 (0.0)	6 (16.2)		5 (23.8)	7 (38.9)	
AC	7 (77.8)	24 (64.9)		13 (61.9)	6 (33.3)	
AA	2 (22.2)	7 (18.9)		3 (14.3)	5 (27.8)	
AC+AA	9 (100.0)	31 (83.8)	0.327	16 (76.2)	11(61.1)	0.309
Alleles			0.757			0.944
C	7 (38.9)	36 (42.9)		23 (54.8)	20 (55.6)	
A	11 (61.1)	48 (57.1)		19 (45.2)	16 (44.4)	
*VDR-Fokl genotypes*			0.033 *			0.646
TT	0 (0.0)	9 (24.3)		9 (42.9)	9 (50.0)	
CT	4 (44.4)	22 (59.5)		9 (42.9)	8 (44.4)	
CC	5 (55.6)	6 (16.2)		3 (14.2)	1 (5.6)	
CT+CC	9 (100.0)	28 (75.7)	0.171	12 (57.1)	9 (50.0)	0.656
Alleles			0.015 *			0.454
T	4 (22.2)	40 (54.1)		27 (64.3)	26 (72.2)	
C	14 (77.8)	34 (45.9)		15 (35.7)	10 (27.8)	
VDR-TaqI *genotypes*			0.514			0.612
CC	0 (0.0)	5 (13.5)		2 (9.5)	3 (16.7)	
CT	4 (44.4)	19 (51.4)		10 (47.6)	6 (33.3)	
TT	5 (55.6)	13 (35.1)		9 (42.9)	9 50.0)	
CT+TT	9 (100.0)	32 (86.5)	0.566	19 (90.5)	15 (83.3)	0.506
Alleles			0.178			
C	4 (22.2)	29 (39.2)		14 (33.3)	12 (33.3)	1.000
T	14 (77.8)	45 (60.8)		28 (66.7)	24 (66.7)	

SHBG: hormone-binding globulin; DHEA-S: dehydroepiandrosterone sulfate; MDA: malondialdehyde oxidative stress; data presented as ^(a)^ arithmetic mean (sample standard deviation) or ^(b)^ geometric mean (geometric standard deviation) or ^(c)^ median [25th percentile, 75th percentile] or n = number of subjects; *p*-values obtained from Mann–Whitney test, Chi-squared test, Fisher’s exact test or Student’s *t*-test with equal variances applied on transformed data on logarithmic scale; * significant result: *p*-value < 0.05.

**Table 4:** Distributions values of hormonal and oxidative stress characteristics in dominant genotype model for vitamin D receptor gene polymorphisms (FokI, ApaI, TaqI) in PCOS patients.

Characteristics	Women without PCOS			Women with PCOS		
*VDR-ApaI genotypes*	CC (n_1_ = 12)	AC+AA (n_2_ = 27)	*p*-Value	CC	AC+AA	*p*-Value
Testosterone total (nmol/L) ^(a)^	1.37 (0.55)	1.52 (0.46)	0.367	1.31 (0.77)	1.46 (0.36)	0.654
SHBG (nmol/L) ^(b)^	59.22 (1.32)	63.61 (1.59)	0.489	48.85 (1.33)	47.82 (1.66)	0.921
DHEA-S (lg/dL) ^(c)^	0.89 [0.64, 1.10]	1.02 [0.84, 1.19]	0.207	1.30 [1.30, 1.45]	1.15 [0.98, 1.41]	0.473
MDA (nmol/mL) ^(c)^	1.30 [1.21, 1.59]	1.20 [1.08, 1.33]	0.094	1.90 [1.61, 2.11]	1.90 [1.62, 2.33]	0.870
*VDR-Fokl genotypes*	TT (n_1_ = 18)	CT+CC (n_2_ = 21)		TT (n_1_ = 9)	CT+CC (n_2_ = 37)	
Testosterone total (nmol/L) ^(a)^	1.54 (0.49)	1.41 (0.46)	0.407	1.64 (0.34)	1.39 (0.43)	0.105
SHBG (nmol/L) ^(b)^	69.24 (1.56)	55.75 (1.45)	0.107	50.65 (1.53)	47.32 (1.64)	0.708
DHEA-S (lg/dL) ^(c)^	0.98 [0.78, 1.09]	0.92 [0.83, 1.20]	0.977	1.14 [1.06, 1.21]	1.17 [0.96, 1.47]	0.967
MDA (nmol/mL) ^(c)^	1.18 [1.02, 1.27]	1.29 [1.19, 1.45]	0.016*	2.15 [1.92, 2.43]	1.87 [1.57, 2.18]	0.054
VDR-TaqI *genotypes*	CC (n_1_ = 5)	CT+TT (n_1_ = 34)		CC (n_1_ = 5)	CT+TT (n_1_ = 41)	
Testosterone total (nmol/L) ^(a)^	1.48 (0.52)	1.47 (0.49)	0.979	1.35 (0.72)	1.45 (0.39)	0.776
SHBG (nmol/L) ^(b)^	51.04 (1.48)	63.34 (1.52)	0.285	60.84 (1.90)	46.58 (1.58)	0.245
DHEA-S (lg/dL) ^(c)^	1.06 [0.92, 1.22]	0.96 [0.76, 1.16]	0.425	1.19 [0.92, 1.47]	1.16 [0.99, 1.38]	0.986
MDA (nmol/mL) ^(c)^	1.33 [1.23, 1.57]	1.24 [1.10, 1.36]	0.231	1.99 [1.92, 2.15]	1.87 [1.57, 2.33]	0.323

SHBG: hormone-binding globulin; DHEA-S: dehydroepiandrosterone sulfate; MDA: malondialdehyde oxidative stress; data presented as ^(a)^ arithmetic mean (sample standard deviation) or ^(b)^ geometric mean (geometric standard deviation) or ^(c)^ median [25th percentile, 75th percentile] or n = number of subjects; *p*-values obtained from Mann–Whitney test, or Student’s *t*-test with equal variances applied on transformed data on logarithmic scale; * significant result: *p*-value < 0.05.

## Data Availability

All data generated in this study was reported in the paper.

## Supplementary Materials

The following supporting information can be downloaded at: https://www.mdpi.com/article/10.3390/medicina60091501/s1. Table S1. Demographic, hormonal, stress oxidative characteristics, genotypes, and alleles of Vitamin D receptor gene polymorphisms (FokI, ApaI, TaqI) in PCOs patients with and without hirsutism. Table S2. Demographic, hormonal, stress oxidative characteristics, genotypes, and alleles of Vitamin D receptor gene polymorphisms (FokI, ApaI, TaqI) in PCOs patients with and without androgenic alopecia.
