# Supplementary material for: The Impact of Vitamin D Receptor Gene Polymorphisms (FokI, ApaI, TaqI) in Correlation with Oxidative Stress and Hormonal and Dermatologic Manifestations in Polycystic Ovary Syndrome
Source: Medicina (Kaunas). 2024 Sep 14;60(9):1501. doi: 10.3390/medicina60091501 (PMC11433945; doi:10.3390/medicina60091501)
Supplement: Supplementary file 1 [file medicina-60-01501-s001.zip › medicina-3187323-supplementary.pdf]

Table S1. Demographic, hormonal, stress oxidative characteristics, genotypes, and alleles of Vitamin D receptor gene polymorphisms (FokI, ApaI, TaqI) in PCOs patients with and without hirsutism.

Characteristics	PCOS without Hirsutism (n_1 = 19)	PCOS with Hirsutism (n_2 = 27)	p-value
Age (years) [(1]	25.58 (2.61)	24.78 (2.78)	0.329
BMI (kg/m^2) [(2]	22.41 (1.15)	22.05 (1.18)	0.728
Testosterone total (nmol/L) [(1]	1.36 (0.44)	1.49 (0.41)	0.286
SHBG (nmol/L) [(2]	45.27 (1.58)	49.94 (1.65)	0.501
DHEA-S (lg/dL) [(3]	0.99[0.72, 1.63]	1.19 [1.08, 1.37]	0.199
MDA (nmol/ml) [(3]	1.88 [1.60, 2.34]	1.92 [1.62, 2.16]	0.746
VDR- ApaI genotypes			0.289
CC	1 (5.3)	5 (18.5)	
AC	15 (78.9)	16 (59.3)	
AA	3 (15.8)	6 (22.2)	
AC+AA	18 (94.7)	22 (81.5)	0.377
Alleles			0.747
C	17 (44.7)	26 (48.1)	
A	21(55.3)	28 (51.9)	
VDR- Fokl genotypes			0.473
TT	2 (10.5)	7 (25.9)	
CT	12 (63.2)	14 (51.9)	
CC	5 (26.3)	6 (22.2)	
CT+CC	17 (89.5)	20 (74.1)	0.270
Alleles			
T	16 (42.1)	28 (51.9)	0.357
C	22 (57.9)	26 (48.1)	
**VDR- TaqI *genotypes***			0.586
CC	1 (5.3)	4 (14.8)	
CT	11 (57.9)	12 (44.4)	
TT	7 (36.8)	11(40.7)	
CT+TT	18 (93.7)	23 (85.1)	0.387
Alleles			0.781
C	13 (34.2)	20 (37.0)	
T	25 (65.8)	34 (63.0)	

DHEA-S: dehydroepiandrosterone sulfate; MDA: Malondialdehyde oxidative stress; data presented as [(1] arithmetic mean (sample standard deviation) or [(2] geometric mean (geometric standard deviation) or [(3] median [25th percentile, 75th percentile] or n = number of subjects; p-values obtained from Mann-Whitney test, Chi-squared test, Fisher's exact test or Student-t test with equal variances applied on transformed data on logarithmic scale; [*] significant result: p-value <0.05.

Table S2. Demographic, hormonal, stress oxidative characteristics, genotypes, and alleles of Vitamin D receptor gene polymorphisms (FokI, ApaI, TaqI) in PCOs patients with and without androgenic alopecia.

Characteristics	PCOS without Alopecia (n_1 = 36)	PCOS with Alopecia (n_2 = 10)	p-value
Age (years) [(1]	25.06 (2.65)	25.30 (3.06)	0.804
BMI (kg/m^2) [(2]	22.19 (1.16)	22.23 (1.19)	0.969
Testosterone total (nmol/L) [(1]	1.41 (0.42)	1.53 (0.44)	0.446
SHBG (nmol/L) [(3]	48.29 (1.65)	46.78 (1.52)	0.856
DHEA-S (lg/dL) [(a]	1.17 [0.99, 1.37]	1.32 [1.00, 1.95]	0.351
MDA (nmol/ml) [(a]	1.87 [1.57, 2.18]	2.15 [1.89, 2.41]	0.122
VDR- ApaI genotypes			0.659
CC	5 (13.9)	1 (10.0)	
AC	25 (69.4)	6 (60.0)	
AA	6 (16.7)	3 (30.0)	
AC+AA	31 (86.1)	9 (90.0)	1.000
Alleles			0.495
C	35 (48.6)	8 (40.0)	

A	37 (51.4)	12 (60.0)	
VDR- Fokl genotypes			0.629
TT	6 (16.7)	3 (30.0)	
CT	21 (58.3)	5 (50.0)	
CC	9 (25.0)	2 (20.0)	
CT+CC	30 (83.3)	7 (70.0)	0.384
Alleles			
T	33 (45.8)	11 (55.0)	0.468
C	39 (54.2)	9 (45.0)	
**VDR- TaqI *genotypes***			0.773
CC	4 (11.1)	1 (10.0)	
CT	19 (52.8)	4 (40.0)	
TT	13 (36.1)	5 (50.0)	
CT+TT	32 (88.9)	9 (90.0)	1.000
Alleles			0.536
C	27 (37.5)	6 (30.0)	
T	45 (62.5)	14 (70.0)	

DHEA-S: dehydroepiandrosterone sulfate; MDA: Malondialdehyde oxidative stress; data presented as (1 arithmetic mean (sample standard deviation) or (2 geometric mean (geometric standard deviation) or (3 median [25th percentile, 75th percentile] or n = number of subjects; p-values obtained from Mann-Whitney test, Chi-squared test, Fisher's exact test or Student-t test with equal variances applied on transformed data on logarithmic scale.
